# Prevalence and factors associated with double and triple burden of malnutrition among mothers and children in Nepal: evidence from 2016 Nepal demographic and health survey

**DOI:** 10.1186/s12889-020-8356-y

**Published:** 2020-03-29

**Authors:** Dev Ram Sunuwar, Devendra Raj Singh, Pranil Man Singh Pradhan

**Affiliations:** 1 Department of Nutrition and Dietetics, Nepal APF Hospital, Kathmandu, Nepal; 2 Department of Public Health, Asian College for Advance Studies, Purbanchal University, Lalitpur, Nepal; 3 Southeast Asia Development Actions Network (SADAN), Lalitpur, Nepal; 4 Department of Community Medicine, Maharajgunj Medical Campus, Institute of Medicine, Tribhuvan University, Kathmandu, Nepal

**Keywords:** Prevalence, Double burden, Triple burden, Malnutrition, Nepal

## Abstract

**Background:**

Malnutrition in mothers and children is a significant public health challenge in developing countries such as Nepal. Although undernutrition in children has been gradually decreasing, the coexistence of various forms of malnutrition in mothers and children has continued to rise globally. There is a gap in knowledge of the coexistence of such multiple burdens of malnutrition in the Nepalese context. The aims of this study were to explore the coexistence of various forms of malnutrition and associated factors among mother-child pairs residing in the same household.

**Methods:**

A total sample of 2261 mother-child pairs from the Nepal Demographic and Health Survey (NDHS) 2016 were included in the study. Anthropometric measurements and hemoglobin levels of children and anthropometric measurements of their mothers were collected. Bivariate and multivariable logistic regression models were used to assess the factors associated with the double burden of malnutrition (DBM) and the triple burden of malnutrition (TBM).

**Results:**

Prevalence of DBM and TBM was 6.60% (95% CI: 5.13–8.84) and 7.00% (95% CI: 5.42–8.99) respectively in the same households. In the adjusted multivariable logistic regression models, mothers with short stature (AOR = 4.18, 95% CI: 2.04–8.52), from the richest wealth quintile (AOR = 2.46, 95% CI: 1.17–5.15), aged over 35 years (AOR = 3.08, 95% CI: 1.20–7.86), and those who had achieved at least secondary level education (AOR = 2.05, 95% CI: 1.03–4.07) were more likely to suffer from the DBM. Similarly, mothers with short stature (AOR = 5.01, 95% CI: 2.45–10.24), from the richest wealth quintile (AOR = 2.66, 95% CI: 1.28–5.54), aged over 35 years (AOR = 3.41, 95% CI: 1.26–9.17), and those who had achieved at least secondary level education (AOR = 2.05, 95% CI: 1.00–4.18) were more likely to suffer from the TBM.

**Conclusions:**

Overall, there is a low prevalence of double and triple burden of malnutrition among mother-child pairs in Nepal. Older mothers with short stature and those from richer wealth quintiles were more likely to suffer from double and triple burden of malnutrition.

## Introduction

The various forms of malnutrition among children and mothers are significant public health challenges in low- and middle-income countries [1]. Various forms of malnutrition contribute to the double and triple burden of malnutrition. The double burden of malnutrition (DBM) is defined by the coexistence of maternal overweight and obesity along with child undernutrition within the same household level [2, 3]. Triple burden of malnutrition (TBM) refers to the coexistence of overnutrition, undernutrition and micronutrient deficiencies [4, 5]. Overnutrition, undernutrition, and micronutrient deficiencies equally increase the risk of various health problems [6]. Child undernutrition increases the risk of childhood mortality and poor cognitive development [7], and overnutrition is associated with increased risk of various non-communicable diseases such as high blood glucose levels, raised blood pressure, abdominal obesity and high lipid profiles [8]. Overweight/obesity during pregnancy is positively linked with several adverse maternal and fetal consequences during pregnancy, delivery and the postpartum period [9, 10].

Globally, the prevalence of undernutrition (stunting, wasting and underweight) in children has declined from an estimated 40% in 1990 to an estimated 26% in 2011, with an average annual rate of reduction of 2.1% per year. At the same time, maternal overweight has increased from an estimated 20% in 1990 to an estimated 30% in 2008, whilst maternal obesity also increased from an estimated 5% in 1990 to an estimated 10% in 2008 [6]. Furthermore, the prevalence of overweight and obesity has been projected to increase by two-thirds in South and Southeast Asia by 2030 [9]. Among South and Southeast Asian countries, the prevalence of overweight and obesity among women of reproductive age was estimated to be 21.3 and 8.6% respectively [9]. In Nepal, a systematic review based on a nationally representative report from 2001 to 2016, showed that the prevalence of stunting, wasting, and underweight among children has declined from 57.2 to 35.8%, 11.2 to 9.7%, and 42.7 to 27.0% respectively. However, overweight and obesity among women has increased from 6.5 to 22.1% over the same period [11]. The prevalence of stunting, wasting, and underweight has declined in the last decade in the context of Nepal [11], however, anemia in children aged under 5 years has been stagnant. Overweight and obesity has increased in all women acrossall socio-demographic groups [12, 13]. A nationally representative Demographic and Health Survey and National Health and Nutrition survey from different low and middle-income countries reported that overweight and obesity in mothers was found to coexist with stunting, wasting and underweight among children within the same households [14, 15].

Association of socioeconomic status (SES) and the double burden of malnutrition has been explored in several studies [14, 16]. Maternal overweight/obesity and child undernutrition among mother-child pairs is thought to be the result of an interaction of changes related to socio-demographic and economic status, dietary habit and intensity of physical activity [17]. Popkin et al. (2012) has highlighted that most low- and middle-income countries are undergoing economic and nutrition transition [18]. In addition, various studies have also indicated that the double burden of malnutrition is associated with older mothers, mothers having short stature and a higher level of maternal education and wealth [14, 19, 20]. This might be due to the fact that educated women are more likely to engage in jobs that involve less physical activity, potential dietary differences and economic development [21].. Undernutrition and micronutrient deficiencies are highly prevalent among Nepalese mothers and children under 5 years of age. According to the Nepal Demographic and Health Survey (NDHS), the 2016 report depicted that the prevalence of stunting, wasting, underweight, and anemia among children under 5 years was 35.8, 9.7, 27.0, and 53% respectively.

The coexistence of various forms of malnutrition among mothers and children has continued to rise globally [22]. To our knowledge, overnutrition, undernutrition and micronutrient deficiencies in mother-child pairs within the same household has not yet been explored using nationally representative data in Nepal. This study aims to provide important evidence for the prevalence of double and triple burden of malnutrition and associated factors among mother-child pairs in the Nepalese context.

## Methods

### Study design and population

This study utilized secondary data from the Nepal Demographic and Health Survey (NDHS) 2016, a nationally representative cross-sectional survey, to explore the prevalence of double and triple burden of malnutrition and associated factors among mother-child pairs. This survey was carried out as part of the DHS program by New ERA under the guidance of the Ministry of Health, Government of Nepal and supported by ICF international and United States Agency for International Development (USAID). The study population for this study was mother-child pairs from the Nepal Demographic and Health Survey 2016.

### Sampling strategy

**Fig. 1:**
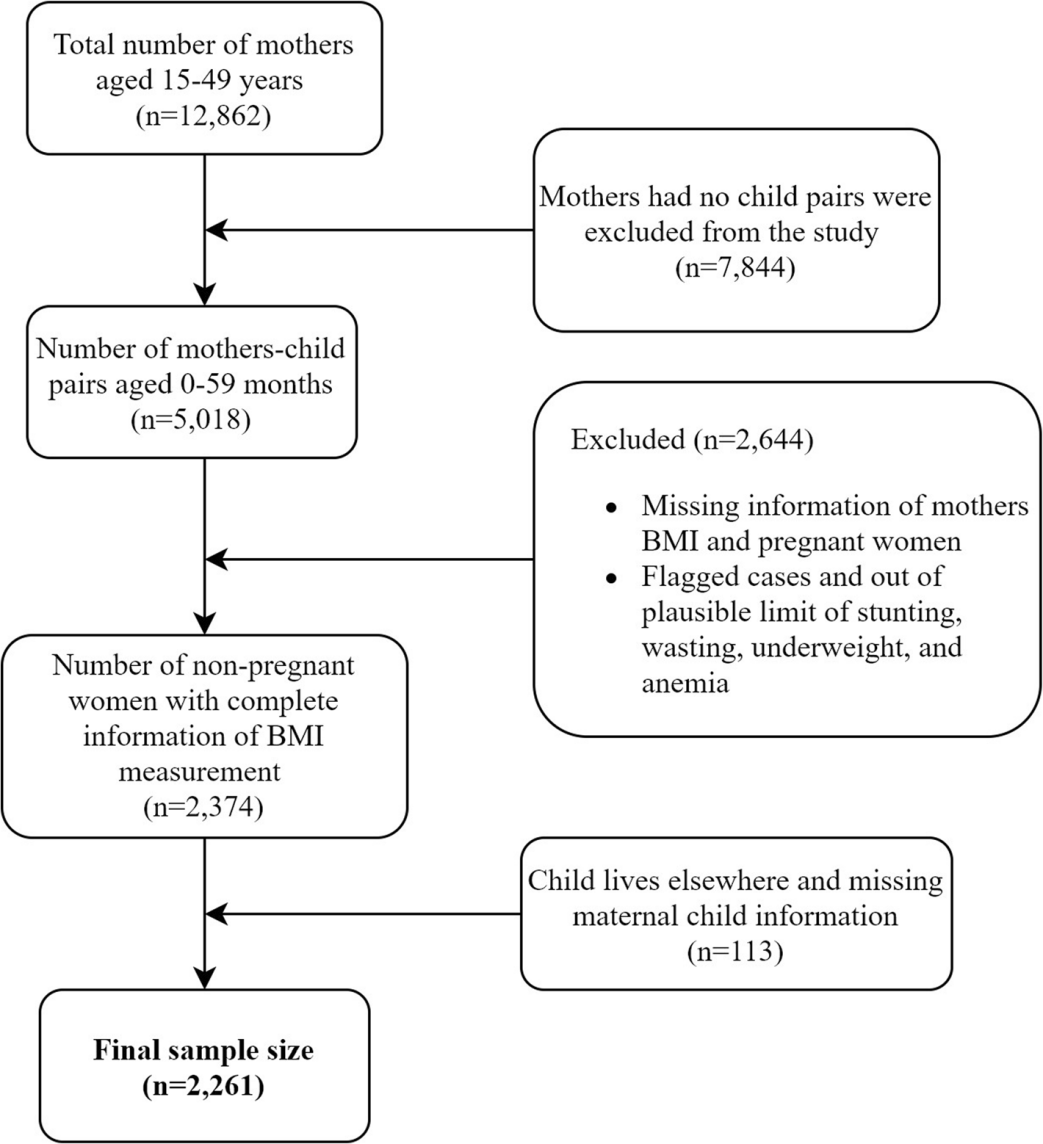
Flow chart for sample size selection

The NDHS 2016 utilized a stratified, two-stage cluster sampling design to provide representative estimates for seven provinces, three ecological zones, and urban and rural areas. The survey used enumeration areas (EAs) which is a primary sampling unit (PSU) and was selected from 383 wards in both rural (*n* = 199) and urban (*n* = 184) areas with probability proportional to size method. In the second stage, 30 households on average within each EA were selected using a systematic sampling technique. A more detailed methodology of the NDHS has been published in the most recent NDHS report [13]. The details of the sample size and exclusion criteria for the selection of the mother-child pairs are presented in Fig. 1.

### Data collection techniques

In this study, we used anthropometric and biochemical indices such as height-for-age, weight-for-height, and weight-for-age and hemoglobin levels to evaluate the nutritional status of each child aged 0–59 months. The WHO Multicenter Growth Reference Study Group, 2006 was used to calculate the anthropometric indicators to evaluate the nutritional status of each child [23]. Children suffering from stunting, wasting and underweight were defined as children with Z-scores below − 2 standard deviation (more than 2 standard deviations below the reference median), for height-for-age (HAZ), weight-for-height (WHZ) and weight-for-age (WAZ) respectively. We categorized the blood hemoglobin level as anemic (< 11 g/dl) and not anemic (≥11 g/dl) for the purpose of analysis. Similarly, we used body mass index (BMI) classification according to WHO for mothers aged 15–49 years. The standard WHO cut-off value was used to determine the normal BMI (18.5 to < 24.99 kg/m^2^) and overweight/obesity (> 25.0 kg/m^2^) [24].

### Study variables

The detailed plan for data coding and description of the study variables is given in Table 1.

**Table 1:** Plan for data coding and description of the study variables

Study variables	Coding category for analysis
**Outcome variables**	
Stunting (HAZ)	0 = Normal HAZ/not stunted (HAZ -2SD and above) 1 = stunted (HAZ < -2SD)
Wasting (WHZ)	0 = Normal WHZ/not wasted (WHZ-2SD to +2SD) 1 = Wasted (WHZ < -2SD)
Underweight (WAZ)	0 = Normal WAZ/not underweight (WHZ-2SD and above) 1 = underweight (WAZ < -2SD)
Child anemia	0 = Normal/not anemic (hemoglobin level > 11 g/dl), 1 = anemic (hemoglobin level < 11 g/dl)
Mothers BMI (Continuous, calculated using measured height and weight)	0 = Normal (18.5–24.9 kg/m^2^) 1 = Overweight/Obese (≥25 kg/m^2^)
Double burden of malnutrition (DBM):overweight/obese mothers was paired with her child having one form of undernourished (stunted or wasted or underweight)	0 = Normal or not overweight/obese mother and not undernourished child (stunted or wasted or underweight) 1 = overweight/obese mother and undernourished child (stunted or wasted or underweight)
Triple burden of malnutrition (TBM):overweight/obese mothers was paired with her child having one form of undernourished (stunted or wasted or underweight) plus anemic child	0 = Normal or not overweight/obese mother and not undernourished child (stunted or wasted or underweight) plus not anemic child 1 = overweight/obese mother and undernourished child (stunted or wasted or underweight) plus anemic child

### Outcome variables

In order to simplify the analysis of the outcome variables, we dichotomized all dependent variables into presence of malnutrition (coded 1), versus absence (coded 0). We created four different categories of malnutrition such as overweight/obese mother and stunted child (OM/SC), overweight/obese mother and wasted child (OM/WC), overweight/obese mother and underweight child (OM/UC), overweight/obese mother and anemic child (OM/AC) at the same household level. Four different categories were further combined to form two categories: overweight/obesity mother and undernourished child (stunting or wasting or underweight) which was considered as the double burden of malnutrition (DBM) [25] and the double burden of malnutrition plus anemic child (DBM + anemia) was regarded as the triple burden of malnutrition (TBM) [4, 5].

### Independent variable

In this study, we included maternal socio-demographic factors (mother’s age, age at first birth, ethnicity, place of residence, province, education level, occupation, household wealth status, height, iron/folate intake, antenatal care (ANC) visits, parity, delivery by cesarean section), fathers occupation, education and child factors (age of child, child sex, vitamin A consumption, deworming tablet consumption, breastfeeding status, weight at birth, and total number of children ever born from single mother) as independent variables.

### Data analysis

Data were analyzed using STATA/MP version 14.1 (StataCorp LP, College Station, Texas). The ‘svy’ command was used to adjust for EAs and disproportionate sampling weight and non-response. The datasets for women and child files were merged. The prevalence of overweight/obese mother and stunted child (OM/SC), overweight/obese mother and wasted child (OM/WC), overweight/obese mother underweight child (OM/UC), overweight/obese mother and anemic child (OM/AC), double and triple burden of malnutrition were presented as weighted percentages with 95% confidence intervals. The bivariate and multivariable logistic regression models were performed to assess the factors associated with the double and triple burden of malnutrition. To prevent statistical bias in the multivariable logistic regression model, we examined and reported multicollinearity among the predictor variables using variation inflation factors (VIF). In this study, we used “10” as a cut-off value for the maximum level of VIF [26]. Bivariate analysis was performed to assess the association of socio-demographic factors with outcome variables. All variables with statistically significant associations (*p* < 0.05) in bivariate analysis were included in the multivariable regression model. Results were presented as crude odds ratio (COR) and adjusted odds ratio (AOR) with 95% confidence intervals (CI). *P*-value < 0.05 was considered as statistically significant.

### Ethical considerations

This study was a secondary analysis of the NDHS 2016 data, thus no separate ethical approval was required. However, ethical clearance for the NDHS was obtained from the ethical review board of Nepal Health Research Council and the written informed consent was obtained from each participant as per the standard ethical guidelines of the DHS program. We registered and requested for access to data from the DHS website (URL: https://www.dhsprogram.com/data/available-datasets.cfm) and received an approval to access and download the DHS data file.

## Results

A total of 2261 mother-child pairs were included in the study (Fig. 1). Table 2 shows socio-demographic information of the participants and different forms of malnutrition existing among mother-child pairs in the same households in Nepal. The mean (+SD) age of the mothers and age of the child was 26.36 (+ 5.64) years and 29.01 (+ 17.37) months respectively. Approximately half of mothers (49.22%) were in the 25–34 years age group and more than half of mothers (52.77%) were below 19 years of age at the time of their first birth. One-third of mothers (33.68%) and only 15.12% of fathers did not receive any formal education and approximately one-third of mothers (32.36%) and more than one-third of fathers (43.86%) attained secondary level education. The majority of mothers (46.69%) and only 19.32% of the fathers were involved in agriculture. One-qurter of mothers (25.89%) were living in province number 2, more than one-third of mothers (42.04%) belonged to poor wealth status family and the majority of mothers (90%) had normal height. Slightly less than one-quarter of childrens (22.53%) were below 12 months of age groups, more than half of children (52.71%) were male, more than two-thirds of the child (74.52%) received a vitamin A capsule in the previous 6 months and more than half of the child (59.82%) were taking deworming treatment. Approximately two-thirds of children (67.72%) were born with an average birth weight.

**Table 2:** Socio-demographic characteristics of the study participants (*N* = 2261)

Variables		Mean **+** SD
**Mother’s age (years)**		26.36 + 5.64
**Child’s age (months)**		29.01 + 17.37
**Mother’s age at 1st birth (year)**		19.83 + 3.34
**Maternal factors**	**characteristics**	**n(%)**^**a**^
**Age groups (years**)	15–24	951 (41.14)
	25–34	1104 (49.22)
	> 35	206 (9.64)
**Age at 1st birth (years)**	< 19	1190 (52.77)
	20–29	1039 (45.73)
	> 30	32 (1.50)
**Province**	Province 1	305 (16.27)
	Province 2	467 (25.89)
	Province 3	227 (15.61)
	Province 4	232 (8.01)
	Province 5	382 (18.93)
	Province 6	341 (6.57)
	Province 7	307 (8.71)
**Education**	No education	744 (33.68)
	Primary	423 (19.53)
	Secondary	744 (32.36)
	higher	350 (14.42)
**Occupation**	Agriculture	1120 (46.69)
	No job	855 (40.35)
	Services	286 (12.95)
**Wealth status**	Poor	1052 (42.04)
	Middle	476 (22.02)
	Rich	733 (35.94)
**Height**	Normal height	2046 (90)
	Short stature	215 (10)
**Delivery by CS**	No	2071 (90.20)
	Yes	190 (9.80)
**Father’s occupation**	Agriculture	422 (19.32)
	No job	952 (41.9)
	Services	869 (38.78)
**Father’s education**	No education	310 (15.12)
	Primary	509 (22.79)
	Secondary	1000 (43.86)
	higher	422 (18.24)
**Child factors**		
**Child’s age (months)**	< 12 months	514 (22.53)
	13–23 months	435 (19.18)
	24–35 months	427 (18.45)
	36–47 months	452 (20.11)
	48–59 months	433 (19.73)
**Child’s sex**	Male	1193 (52.71)
	Female	1068 (47.29)
**Vitamin A**	Yes	1688 (74.52)
	No	573 (25.48)
**Deworming**	Yes	1399 (59.82)
	No	862 (40.18)
**Currently breastfeeding**	Yes	1824 (78.80)
	No	437 (21.20)
**Birth weight**	Average	1510 (67.72)
	Large	350 (15.72)
	Small	396 (16.61)
**OM/SC (*n*** **= 1027)**	Not stunted	949 (91.70)
	Stunted	78 (8.30)
**OM/UC(*n*** **= 1138)**	Not underweight	1100 (96.63)
	Underweight	38 (3.37)
**OM/WC(*n*** **= 1350)**	Not wasted	1335 (98.75)
	Wasted	15 (1.25)
**OM/AC(*n*** **= 751)**	Not anemic	625 (81.11)
	Anemic	126 (18.89)
**DBM(*n*** **= 1529)**	Normal	1437 (93.4)
	OWOBM/UC	92 (6.60)
**TBM(*n*** **= 1381)**	Normal	1293 (93)
	OWOBM/UC/AC	88 (7.00)

^a^ Frequency are unweighted; percentage are weighted

*OM/SC* Overweight/obese mother and stunted child

*OM/WC* Overweight/obese mother and wasted child

*OM/UC* Overweight/obese mother and underweight child

*OM/AC* Overweight/obese mother and anemic child

*DBM* Double burden of malnutrition (Overweight/obese mother and undernourished child (stunted or wasted or underweight) at the same household

*TBM* Triple burden of malnutrition (Overweight/obese mother and undernourished and anemic child) at the same household

*CS* Caesarean section

The prevalence of an overweight/obese mother and a stunted child (OM/SC) was 8.30% (95% CI: 6.32–10.84), overweight/obese mother and wasted child (OM/WC) was 1.25% (95% CI: 0.74–2.11), overweight/obese mother and underweight child (OM/UC) was 3.37% (95% CI: 2.34–4.83) and overweight/obese mother and anemic child (OM/AC) was 18.89% (95% CI: 15.43–22.83). The prevalence of the DBM was 6.60% (95% CI: 5.13–8.84) and TBM was 7.00% (95% CI: 5.42–8.99) at the household level.

Table 3. Depicts bivariate and multivariate logistic regression models for the different forms of malnutrition and their associated factors among mother-child pairs. The following results are the interpretation of different forms of malnutrition and associated factors.

**Table 3:** Bivariate and multivariable analysis of double and triple burden of malnutrition among mother-child pairs and its associated factors (*n* = 2261)

Maternal factors	DBM		TBM	
	COR(95% CI)	AOR(95% CI)^b^	COR(95% CI)	AOR(95% CI)^c^
**Age group**				
15–24	1	1	1	1
25–34	1.61 (0.87–2.98)	1.59 (0.83–3.03)	1.64 (0.88–3.08)	1.96 (1.04–3.71)^*^
> 35	2.59 (1.13–5.94)^**^	3.08 (1.20–7.86)^**^	3.11 (1.34–7.22)^**^	3.41 (1.26–9.17)^**^
**Age at 1st birth**				
< 19 years	0.13 (0.03–0.45)^**^		0.16 (0.04–0.62)^**^	0.34 (0.66–1.83)
20–29 years	0.12 (0.03–0.44)^**^		0.16 (0.03–0.64)^**^	0.18 (0.03–1.06)
Above 30 years	1		1	1
**Province**				
Province 1	0.89 (0.40–2.00)	1.20 (0.50–2.85)	0.89 (0.38–2.03)	1.08 (0.47–2.49)
Province 2	0.08 (0.02–0.29)^***^	0.13 (0.03–0.47)^***^	0.10 (0.03–0.33)^***^	0.11 (0.03–0.41)^***^
Province 3	1	1	1	1
Province 4	0.73 (0.31–1.71)	0.97 (0.36–2.61)	0.71 (0.29–1.70)	0.85 (0.31–2.32)
Province 5	0.51 (0.21–1.22)	0.65 (0.25–1.72)	0.57 (0.23–1.38)	0.65 (0.27–1.59)
Province 6	0.24 (0.07–.75)^*^	0.47 (0.14–1.58)	0.28 (0.09–0.87)^*^	0.50 (0.15–1.67)
Province 7	0.13 (0.04–0.41)^***^	0.24 (0.07–0.86)^*^	0.14 (0.04–0.45)^***^	0.23 (0.06–0.82)^*^
**Education**				
No education	1	1	1	1
Primary	1.14 (0.55–2.34)	1.04 (0.49–2.22)	1.09 (0.53–2.27)	1.06 (0.46–2.41)
Secondary	2.43 (1.24–4.76)^*^	2.05 (1.03–4.07)^*^	2.09 (1.05–4.16)^*^	2.05 (1.00–4.18)^*^
higher	1.83 (0.81–4.10)	1.04 (0.43–2.49)	1.57 (0.67–3.71)	1.43 (0.53–3.84)
**Occupation**				
Agriculture	1	1	1	1
No job	1.42 (0.75–2.68)	1.19 (0.58–2.42)	1.33 (0.70–2.52)	1.10 (0.53–2.26)
Services	2.82 (1.37–5.81)^*^	1.34 (0.63–2.86)	2.60 (1.26–5.36)^*^	1.33 (0.59–2.96)
**Wealth status**				
Poor	1	1	1	1
Middle	1.15 (0.48–2.75)	1.42 (0.54–3.71)	0.98 (0.38–2.49)	1.61 (0.58–4.40)
Rich	2.89 (1.50–5.54)^***^	2.46 (1.17–5.15)^**^	2.61 (1.36–5.02)^***^	2.66 (1.28–5.54)^***^
**Height**				
Normal height	1	1	1	1
Short stature	3.19 (1.59–6.39)^***^	4.18 (2.04–8.52)^***^	4.38 (2.17–8.86)^***^	5.01 (2.45–10.24)^***^
**Delivery by CS**				
No	1	1	1	
Yes	2.39 (1.18–4.84)^*^	1.44 (0.68–3.04)	1.95 (0.90–4.25)	
**Father’s occupation**				
Agriculture	1		1	
No job	0.98 (0.41–2.32)		1.01 (0.40–2.52)	
Services	1.24 (0.53–2.87)		1.27 (0.52–3.07)	
**Father’s education**				
No education	1		1	
Primary	1.81 (0.58–5.75)		1.84 (0.54–6.17)	
Secondary	1.49 (0.54–4.10)		1.36 (0.46–4.00)	
higher	1.64 (0.56–4.80)		1.51 (0.48–4.71)	
**Child factors**				
**Child’s age**				
< 12 months	1	1	1	1
13–23 months	1.63 (0.80–3.30)	0.85 (0.33–2.20)	2.13 (0.99–4.57)	1.24 (0.47–3.34)
24–35 months	2.15 (1.04–4.46)^*^	1.06 (0.39–2.86)	2.85 (1.33–6.11)^*^	1.60 (0.56–4.57)
36–47 months	2.19 (1.01–4.73)^*^	1.18 (0.43–3.24)	2.75 (1.21–6.24)^*^	1.71 (0.58–5.02)
48–59 months	1.76 (0.79–3.89)	0.77 (0.26–2.26)	2.27 (0.98–5.28)	1.17 (0.36–3.75)
**Child’s sex**				
Male	1		1	
Female	0.84 (0.53–1.33)		0.78 (0.49–1.26)	
**Vitamin A**				
Yes	1	1	1	1
No	0.45 (0.22–0.92)^*^	0.70 (0.29–1.69)	0.40 (0.19–0.86)^*^	0.85 (0.34–2.14)
**Deworming**				
Yes	1	1	1	1
No	0.54 (0.31–0.91)^*^	0.71 (0.32–1.56)	0.49 (0.28–0.86)^*^	0.79 (0.35–1.74)
**Currently breastfeeding**				
Yes	1	1	1	1
No	1.97 (1.10–3.51)^*^	1.34 (0.64–2.79)	1.93 (1.07–3.47)^*^	1.24 (0.59–2.60)
**Birth weight**				
Average	1	1	1	
Large	1.93 (1.13–3.29)^*^	1.57 (0.85–2.92)	1.61 (0.90–2.87)	
Small	1.43 (0.78–2.60)	1.46 (0.77–2.75)	1.53 (0.84–2.81)	

*DBM* Double burden of malnutrition (Overweight/obese mother and undernourished child at the same household)

*TBM* Triple burden of malnutrition (Overweight/obese mother and undernourished and anemic child at the same household)

1: reference category

*COR* Crude odds ratio, *AOR* Adjusted odds ratio

^*^p < 0.05, ^**^p < 0.02, ^***^P < 0.001

^b^ This model was adjusted for mother’s age groups, province, education, occupation, wealth status, mother’s height, delivery by CS, child’s age, vitamin A intake in the last 6 months, deworming, currently breast feeding, and birth weight

^c^ This model was adjusted for mother’s age groups, mother’s age at 1st birth, province, education, occupation, wealth status, mother’s height, child’s age, vitamin A intake in the last 6 months, deworming, and currently breast feeding

### Prevalence and factors associated with the double burden of malnutrition

In the bivariate logistic regression model, several maternal factors were significantly associated with higher odds of the double burden of malnutrition: mother’s short stature compared to normal height (COR = 3.19, 95% CI: 1.59–6.40), mothers from the richest wealth status, compared to poor wealth status (COR = 2.89, 95% CI: 1.50–5.54), mothers who were employed in service occupations, compared to agriculture (COR = 2.82, 95% CI: 1.37–5.81), mothers who had attended at least secondary level of education, compared to no education (COR = 2.43, 95% CI: 1.24–4.76), mothers who had a cesarean section at last delivery, compared to normal delivery (COR = 2.39, 95% CI: 1.18–4.48) and mothers aged over 35 years, compared to 15–24 years (COR = 1.18, 95% CI: 0.38–3.61). In addition, child-related factors that were more likely to increase odds of double burden of malnutrition were as follows: children who were 36–47 months, compared to less than 12 months (COR = 2.19, 95% CI: 1.01–4.73), children with no history of current breastfeeding compared to children being currently breastfed (COR = 1.97, 95% CI: 1.10–3.51) and child’s large size at birth compared to average birth weight (COR = 1.93, 95% CI: 1.13–3.29). Mothers who were 20–29 years of age during first birth of their child compared to above 30 years (AOR = 0.12, 95% CI: 0.03–0.44), mothers living in province number 2 compared to province number 3 (AOR = 0.08, 95% CI: 0.03–0.47), no history of vitamin A intake compared to intake of vitamin A intake among children (COR = 0.45, 95% CI: 0.22–0.92) and no history of deworming compared to deworming among children (COR = 0.54, 95% CI: 0.31–0.91) were found to have lower odds of DBM. Multivariable logistic regression models indicated that mothers with short stature compared to normal height (AOR = 4.18, 95% CI: 2.04–8.52), mothers from richest wealth status compared to poor wealth status (AOR = 2.46, 95% CI: 1.17–5.15), age groups of above 35 years compared to 15–24 years of age groups (AOR = 3.08, 95% CI: 1.20–7.86-4.77), mothers with secondary level of education compared to no education (AOR = 2.05, 95% CI: 1.03–4.07) were more likely to have higher odds of DBM. While mothers living in province number 2 compared to province number 3 (AOR = 0.13, 95% CI: 0.03–0.47), were found to had lower odds of DBM (Table 3).

### Prevalence and factors associated with the triple burden of malnutrition

Bivariate logistic regression model (Table 3) indicated that mother’s short stature compared to normal height (COR = 4.38, 95% CI: 2.17–8.86), mother’s older than 35 years, compared to 15–24 years (COR = 3.11.19, 95% CI: 1.34–7.22), child’s age less than 24–35 months compared to below 12 months (COR = 2.85, 95% CI: 1.33–6.11), had the richest wealth status compared to poor wealth status (COR = 2.61, 95% CI: 1.36–5.02), mothers who worked in services compared to agriculture (COR = 2.61, 95% CI: 1.36–5.02), having at least secondary level of education compared to no education (COR = 2.09, 95% CI: 1.05–4.16), and mothers who had no history of current breastfeeding (COR = 1.93, 95% CI: 1.07–3.47) were more likely to have higher odds of TBM. Likewise, results in the multivariable logistic regression model shows mothers short stature compared to normal height (AOR = 5.01, 95% CI: 2.45–10.24), mothers age groups of above 35 years compared to 15–24 years (AOR = 3.41, 95% CI: 1.26–9.17), mothers from the richest wealth status compared to poor wealth status (AOR = 2.66, 95% CI = 1.28–5.54), and mothers who attended at least secondary level of education compared to no education (AOR = 2.05, 95% CI: 1.00–4.18) were found to have higher odds of TBM. Furthermore, mothers living in province number 2 compared to province number 3 (AOR = 0.11, 95% CI: 0.03–0.41), children with no history of vitamin A intake compared to vitamin A intake (COR = 0.40, 95% CI: 0.19–0.86), and no history of deworming drug intake (COR = 0.49, 95% CI: 0.28–0.86) were found to have lower odds of TBM (Table 3).

## Discussion

This study explored the coexistence of double and triple burden of malnutrition among mother-child pairs within the same household in Nepal. The prevalence of DBM was 6.60% which is higher than that of the neighboring country Bangladesh. A study by Emdadul S et al., [3] found that maternal overnutrition and child undernourishment was 4.9% and Das et al., [25] reported that in Bangladesh the proportion of coexistence of overweight/obese mother and underweight, stunted or wasted child was 6.3%. The double burden of malnutrition was 11% in Indonesia which was higher than most South Asian countries [3, 25, 27]. It has been noted that the overweight/obesity of mothers is associated with the nutrition transition situation that contributes to a positive energy balance which means higher intake of energy-dense food and less energy expenditure [28]. Tendency to consume calorie-dense food with more saturated fat, trans fat and a sedentary lifestyle results in reproductive-aged women gaining weight [18, 27]. In Nepal, the prevalence of overweight/obesity among such populations has increased from an estimated 6.5% in 2001 to 22.1% in 2016 [11].

This study shows that short stature in mothers was strongly associated with the risk of DBM. This result is consistent with that of Oddo et al., [19] who reported that maternal short stature and older age was associated with higher odds of DBM compared to those mothers of normal height and younger age groups. These possible phenomenon could be supported by the findings from Sichieri et al., and Ferreira et al., studies, who reported that BMI gain was significantly higher among short-statured women which reflects malnutrition in early life [29, 30]. Women with short stature were more likely to suffer from chronic degenerative diseases and consequently have undernourished children compared to women of normal stature [30]. Stunting is an intergenerational phenomenon that transfers from mother to child and contributes to small for gestational age (SGA) babies. Malnourished mothesr are more likely to have a low birth weight baby which signifies the importance of exploring the double burden of malnutrition among mother-child pairs [17, 31].

Our results revealed that mothers who were above 35 years were found to be at higher risk of double burden of malnutrition. This result is consistent with Emdadul et al., and Wong et al., who suggested that the prevalence of overweight/obesity was higher in older age groups compared to younger groups [3, 32].

Mothers who attended at least a secondary level of education had a higher risk of experiencing a double burden of malnutrition. This finding is supported by Rai et al., [33] who revealed that women who had primary/secondary levels of education were more likely to be at risk of overweight/obesity. In contrast, various studies have also reported that the double burden of malnutrition is associated with a higher level of maternal education and wealth status [14, 19, 20]. The possible reason could be due to the working environment where educated women are less likely to be involved in physical activity, consequently developing overweight and obesity [21]. Another study suggested that the relationship between education and overweight/obesity is complex and varies from country to country [34]. The explanation for this conflicting finding could be that mothers having a higher level of education may not necessarily be sufficient to adopt behavior change in a healthy lifestyle. In the case of the mothers having with poor health and nutritional knowledge contribute to women being less sensitive to child and her nutritional status or less responsive to health and nutrition issues in terms of food choices and barriers such as food cost, accessibility, availability, lack of cooking skills [17, 35]. As we found that DBM was more prevalent in mothers who had a lower level of education, therefore, providing nutrition education during pregnancy could bridge this nutritional knowledge gap in Nepal [36].

This study found that mothers from province number 2 were less likely to experience DBM compared with mothers from province 3. A possible reason could be that mothers from province number 2 are more likely to be of a lower socioeconomic status and consuming a less diverse diet with an estimated 29% of minimum dietary diversity (MDD) [13] resulting in decreased likelihood of being overweight or obsese (11%) [33, 37]. According to the NDHS 2016 report, the prevalence of overweight/obesity was higher (35%) in province number 3, as well as a higher consumption of diverse food groups with an estimated 64% of MDD. This province is Kathmandu, the largest city (and capital) of the country [37]. Overweight/obesity among mothers could be attributable to the shifting of the Nepalese diet away from locally available staple based foods, to modern fast food and processed food [38], in addition to sedentary lifestyles [33] which contributes to the highest prevalence of overweight and obesity in province number 3.

Our study shows that the prevalence of TBM among mother-child pairs was 7.00% in Nepal. Maternal overweight/obesity and undernourishment in children and its associated factors have been explored in most of Latin American, South and Southeast Asian countries such as Guatemala, Colombia, Brazil, Malaysia, Indonesia, and Bangladesh [3, 19, 25]. However, in mother-child pairs, the coexistence of the triple burden of malnutrition has not yet been examined. Thus, to our knowledge, this study is the first to present the coexistence of overnutrition in mothers with undernutrition and anemia in children residing in the same household. A higher rate of TBM than DBM could be a result of the fact that more than than half (53%) of the children aged 6–59 months were found to be anemic in Nepal in the NDHS [13]. Mamun et al. (2019) also found the prevalence of overweight mothers with anemic children to be 27% in Bangladesh. TBM prevalence is higher, perhaps dueto the higher prevalence of anemia among children aged less than 59 months in developing countries including Nepal. Despite declining undernutrition among children, micronutrient deficiency anemia remains one of the intractable public health problems in South Asia [39]. Despite limited evidence available so far on TBM, maternal overweight/obesity is a crucial factor contributing to child anemia [1]. The plausible mechanism for the phenomenon of TBM has not been examined clearly. A possible reason could be that maternal overweight/obesity is a risk factor for anemia in offspring. Maternal obesity and excessive gestational weight gain pose a increased risk of low neonatal iron status [40]. In obese mothers, impaired iron transfer to the fetus results in lower serum iron as well as transferrin saturation in cord blood as compared to normal-weight mothers [40, 41]. The upregulation of hepcidin under proinflammatory conditions in overweight/obese mothers leads to impaired iron transfer to the placenta resulting in iron deficiency in the newborn [40].

Our study has some limitations. Firstly, the study could not establish a causal pathway of the association between explanatory and dependent variables. Second, data on the outcome measure of maternal overweight/obesity such as dietary intake, physical activity level, health, and nutrition status during pregnancy were not available. Third, the nutritional status of the mother was assessed using BMI only. BMI is less accurate than other methods such as waist-hip ratio, bioelectrical impedance technique, skinfold thickness, and DEXA methods to assess the type of overweight/obesity. Despite these limitations, the strengths of this study were the use of a population-based nationally representative sample. This study provided evidence on the coexistence of overweight/obese mother and undernourished child plus anemia, with associated factors among mother-child pairs in the same household. These findings can provide relevant information to prioritize nutrition intervention programs in Nepal.

## Conclusions

In conclusion, our study revealed a low prevalence of DBM and TBM in Nepal. Our results found that mothers having short stature, mothers from the richest family and older mothers are more prone to the double and triple burden of malnutrition. Also, nation-wide effective implementation of maternal health promotion interventions and nutrition education programs would be a good strategy to prevent overweight/obesity and stunting among children under 5 years of age in Nepal. Likewise, the findings also indicate that wealthier families should not be neglected in prevention strategies of double and triple burden of malnutrition. Nutrition-sensitive and specific interventions need to be scaled up throughout the country for the timely prevention of DBM and TBM among Nepalese mothers and children. Further research is needed to identify the causes and associated risk factors of the double and triple burden of malnutrition which will help pave the way to the sustainable prevention of various forms of malnutrition in Nepal.

## Abbreviations

ANC: Antenatal care
AOR: Adjusted odds ratio
BMI: Body mass index
CI: Confidence interval
COR: Crude odds ratio
DBM: Double burden of malnutrition
DEXA: Dual-energy X-ray absorptiometry
DHS: Demographic and Health Surveys
EAs: Enumeration areas
NDHS: Nepal Demographic and Health Survey
OM/AC: Overweight/obese mother anemic child
OM/SC: Overweight/obese mother stunted child
OM/UC: Overweight/obese mother underweight child
OM/WC: Overweight/obese mother wasted child
PSU: Primary sampling unit
SGA: Small for gestational age
TBM: Triple burden of malnutrition
